# A method based on non-ionizing microwave radiation for ancillary diagnosis of osteoporosis: a pilot study

**DOI:** 10.1186/s12938-022-01038-y

**Published:** 2022-09-22

**Authors:** Gabriela Albuquerque, Agnaldo Cruz, Dionísio Carvalho, Nadja Mayrink, Bruno Pinheiro, Antonio Campos, Josivan Gomes Lima, Jorge Henriques, Ricardo Valentim

**Affiliations:** 1 Advanced Technological Innovation Nucleus—NAVI, Federal Institute of Rio Grande Do Norte, Natal, RN Brazil; 2 Onofre Lopes University Hospital, Natal, Brazil; 3 Department of Informatics Engineering, University of Coimbra, Centre for Informatics and Systems of the University of Coimbra, Coimbra, Portugal

**Keywords:** *Osseus*, Medical device, Osteoporosis, Microwave, Bone mineral density

## Abstract

**Background:**

Osteoporosis is a condition characterized by low bone mineral density, which typically leads to fractures and reduced quality of life. Currently, diagnostic devices used to assess this condition (e.g., dual-energy X-ray absorptiometry) are very costly, making it infeasible to meet the demand for testing in most countries. Therefore, we proposed a preclinical validation of a prototype called *Osseus* in an attempt to enhance osteoporosis screening tests and alleviate their costs. *Osseus* is a device developed to assist bone mineral density classification. It integrates a microcontroller into other peripheral devices to measure the attenuation at the middle phalanx of the middle finger, with two antennas operating at the 2.45 GHz frequency.

**Results:**

We conducted tests with plaster, poultry, and porcine bones. A comparison of the measurements of the original and mechanically altered samples demonstrated that the device can handle the complexity of the tissues within the bone structure and characterize its microarchitecture.

**Conclusions:**

*Osseus* is a device that has been preliminarily validated. Ionising radiation needed for DXA tests is replaced by non-ionising microwave electromagnetic radiation. *Osseus* enables early detection of osteoporosis, reduces costs, and optimizes high-complexity testing referrals. There is a lack of validation studies with the reference/gold standard that are currently under development.

## Introduction

Osteoporosis is often a silent condition that exposes affected individuals to greater fracture risk and consequently hampers their quality of life, e.g., by impairing their mobility and autonomy. For instance, every three seconds, someone suffers an osteoporosis-related fracture in the world [1]. By 2022, the incidence of hip fractures is expected to increase by 310% in men and 240% in women [2], and this proportion significantly increases with age. A study across four Latin American countries [3] estimated the number of fractures in 2018 and 2022 among adults aged 50 to 89 years and the annual cost of osteoporosis in such nations.

**Fig. 1:**
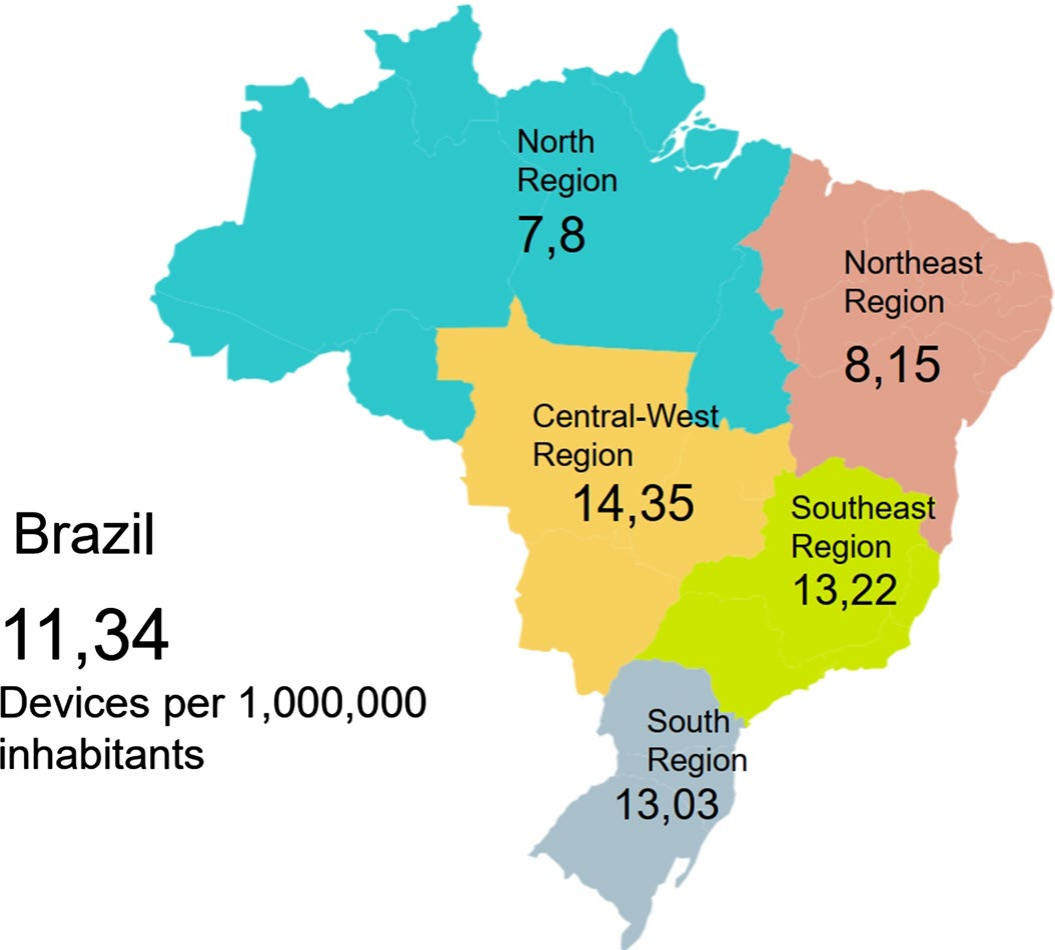
Availability of bone densitometry devices per Brazilian region

By 2022, the number of osteoporotic fractures in Brazil is predicted to increase by 14% compared to 2018 [3]. In addition, the country's yearly cost of the disease is $309,507,247, and medications, tests, hospitalizations, surgeries, and productivity loss are included. Therefore, it is essential to diagnose and treat the disease early to implement preventive measures for mitigating the occurrence of osteoporotic fractures. Disease diagnosis is made using the dual-energy X-ray absorptiometry (DXA) assessment method. It can be applied to the femur, lumbar spine, and forearm [4]. In Brazil, the number of DXA devices in use is 2394 [5], with uneven distribution across the country (Fig. 1). However, such devices are mostly available in large urban centers, and at higher-level health care facilities, so it is insufficient for the entire population to have access. In addition, in public health services, the waiting time for obtaining a bone density screening test is up to 6 months [6].

In addition to DXA, other techniques can also aid in determining bone mineral density (BMD) in clinical research, such as quantitative computed tomography (QCT) and quantitative ultrasound (QUS). The first method, QCT, obtains volumetric bone measurements. However, the radiation dose is substantially higher per scan, and the cost is higher than that of DXA [7]. On the other hand, QUS uses the attenuation and speed of sound to assess BMD at the phalanges of the hand and calcaneus [8]. Albeit radiation-free, QUS devices vary, since there are different measurements for the same skeletal site being assessed—without applying standard diagnostic criteria [9].

Given this scenario, incorporating effective technologies that can promote health and disease prevention is imperative to provide health systems with scientifically sound resoluteness. Incorporating technologies at the Primary Health Care level, from population screening, aims at increasing access to the timely diagnosis of osteoporosis while also allowing, more effectively and swiftly, the adoption of guidelines and interventions at the early onset of the disease.

This study aims to validate a hybrid device, *Osseus*, composed of antennas and developed to perform osteoporosis screening from BMD assessments at the middle phalanx of the middle finger. The analysis was performed by determining the attenuation in the bone tissue. The development of the *Osseus* prototype involved five stages: (1) choice of material, (2) the definition of measures according to the components, (3) creation of embedded software, (4) integration of components, and (5) testing for validation.

Alternative methods involving other skeletal bones have been researched, such as phalanges, to reduce manufacturing costs of devices and improve access to osteoporosis screening. As confirmed in a study, the dielectric constants for healthy and diseased bones in this region are significantly different. This allows the diagnosis of osteoporosis by developing a measurement procedure based on electromagnetic microwave (EM) propagation in the phalanx [10]. This bone region is particularly favorable because it has little muscle tissue, characterized by high attenuation of EM microwaves that propagate through it. In this manner, an electromagnetic signal passing through the phalanx will mostly suffer attenuation due to the bone tissue, which can be higher or lower depending on the bone porosity. To validate this method, we initially conducted tests on animal bones. At this time, validation studies involved only attenuation and its association with bone density.

## Results

### Concept of the work

**Fig. 2:**
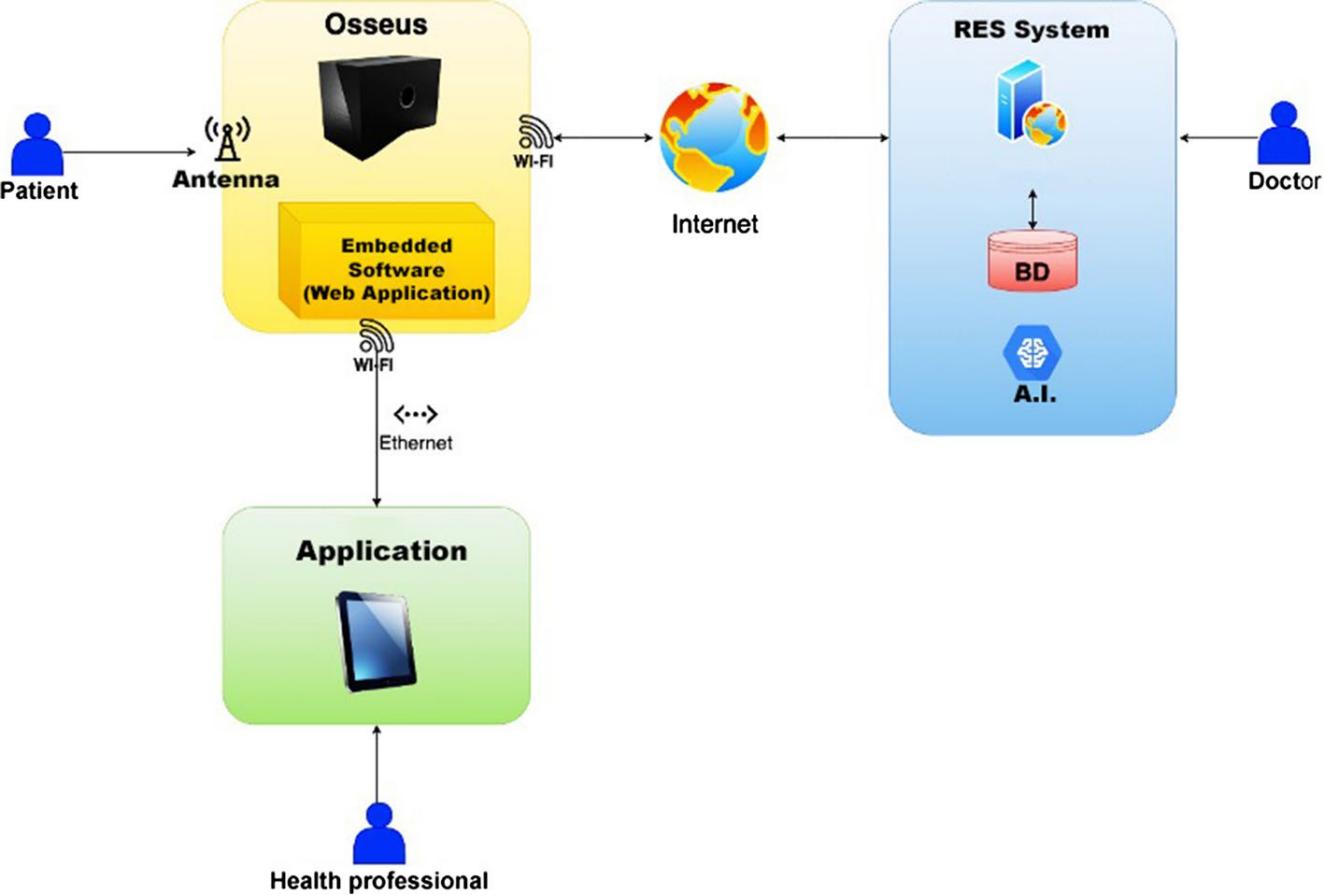
Operation flow of the *Osseus* device

*Osseus* integrates a microcontroller with other peripheral devices to perform the tasks necessary to measure EM attenuation. Figure 2 depicts the flow of the device's operation.

**Fig. 3:**
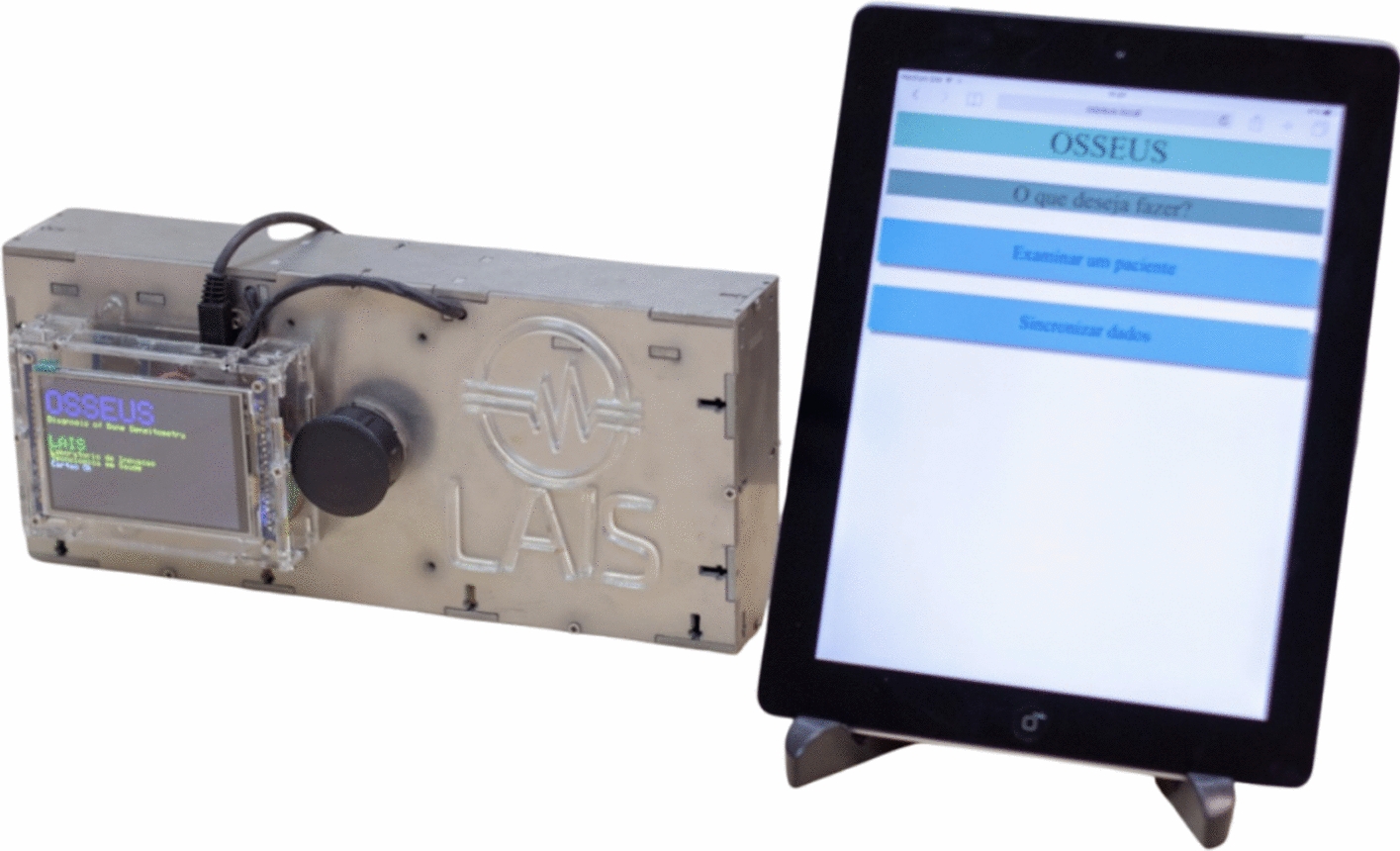
Osseus prototype

The first step of the operation with Osseus starts as an operator fills in the patient's characteristics on an electronic form, then Osseus performs the signal attenuation measurement, with no barrier between two antennas, where the point (frequency of the injected signal) of highest received power is selected so that it can serve as a reference or calibration. After that step, the attenuation test is performed on the patient's finger. Finally, the equipment (Fig. 3) displays the values obtained and sends the data for cloud storage and postprocessing.

The device, through one of its antennas, emits an EM wave in a predefined frequency range. Then, on the opposite side, a similar antenna receives that wave, attenuated by the obstacle corresponding to the patient's finger—specifically, the middle phalanx of the middle finger. A circuit coupled to the antenna identifies the analog signal strength and converts it into a digital signal. Then, based on the patient's characteristics and the signal attenuation, the microcontroller can process the information to indicate whether the individual needs to obtain a DXA scan.

### Experiments performed

Several types of bone tissue were used to prove the functioning of the method used in Osseus. At this time of the research, all samples used in the experiments were tested before and after being artificially drilled with holes to simulate an increase in their porosity.

To obtain the attenuation shown in Table 1, the voltage value measured for each sample is subtracted from the reference value (maximum measured signal without obstacles), which is 1700 mV. For example, as shown in Table 1, sample A1, with the original weight of 204.2 g, caused attenuation of 38.87 mV in relation to the reference value. Just as other samples, A1 was subjected to random mechanical drilling. We used drills of 3, 5, and 7 mm in such a way that the sample structure and mass were altered from their original state. As a result, its weight decreased to 198.6 g, and the sample caused attenuation of 38.67 mV—still in relation to the unhindered signal propagation—yielding a difference of 0.20 mV. In other words, the sample with the lowest amount of bone caused the lowest attenuation. We repeated the process for all samples and found similar results (see Table 1). The measurements in Table 1 show that the equipment uncertainty is 0.01 mV, which gives us an accuracy of 1% with respect to the voltage value measured at the output of the RF power detector.

**Table 1:** Values of attenuation measurements of intact and altered samples

Type of sample	Name	Weight (g)		Attenuation (mV)		Difference (mV)
		Original	Altered	Original	Altered	
Bovine bone	A1	204.2	198.6	38.87	38.67	0.20
	A2	142.9	137.2	39.79	39.52	0.26
	A3	145.3	141.0	40.39	40.31	0.07
	B1	148.3	144.2	43.77	42.86	0.91
	B2	139.3	133.1	40.59	39.96	0.63
	B3	120.6	117.1	38.95	38.81	0.13
	C1	123.0	120.2	39.90	39.88	0.02
	C2	145.7	143.4	41.50	40.89	0.61
	C3	132.6	123.4	40.43	38.95	1.47
Porcine bone	D1	25.1	24.9	118.93	113.36	5.56
	D2	24.7	23.6	78.04	68.10	9.94
	D3	25.1	24.8	56.95	50.62	6.32
	D4	25.6	24.6	82.26	64.47	17.78
Poultry bone	E1	–	–	31.64	25.31	6.32
	E2	–	–	25.31	15.82	9.49
	E3	–	–	15.82	9.49	6.32
	E4	–	–	60.11	28.47	31.64
	E5	–	–	31.64	28.47	3.16
Porcine skin	F1	–	–	155.03	144.15	10.88
Plaster	G1	–	–	50.12	45.76	4.36
Plaster and porcine skin	H1	–	–	208.82	183.51	25.31

The "difference" column in Table 1 does not represent any biophysical meaning, but only the difference between the signal received in an antenna for the situations with the original and altered sample serving as an obstacle for the transmitted signal. However, understanding that a more porous bone tissue causes less attenuation in the transmitted electromagnetic signal when related to reference values ​​that represent healthy or pathological individuals, will represent the ability to classify a patient's bone tissue as normal or with low density.

Attenuation readings with original and altered bone samples (Table 1) show that porcine skin (sample F1) exerts a higher attenuation than a separate plaster cylinder sample (sample G1). This result is positive since it indicates *Osseus* is able to scan human fingers and handle the difference in attenuation caused by human skin. All the other animal bone samples also revealed lower attenuation in the altered samples.

As one can observe, all the differences between readings were positive, which means that all the signal attenuations after sample modifications were lower. The *Osseus* device demonstrated effectiveness in the attenuation measurements, as the attenuation of the signal was recorded in the analyses of the altered samples in all tests (Table 1).

### Ideas for future work

In a second moment, Osseus will carry out tests with real patients, who were previously submitted to a gold standard exam (such as DXA). In this way, the "difference" values ​​obtained will be related to the exam result and also to a series of characteristics of each specific patient (age, gender, finger dimensions, fracture history, etc.) for use in the artificial intelligence algorithms that will be responsible for training Osseus so that it can provide a result on his own, independent of a previous exam.

## Discussion

The first trials of *Osseus* demonstrated effectiveness in measuring bone mineral density in animal models and have proven that it can handle the complexity of the tissues inside bones. A device similar to *Osseus* was developed previously [11], however, it was applied to the forearm, specifically, in the distal region of the radius and the ulna. Its two antennas operate from 30 kHz to 2 GHz band and have a radiofrequency configuration that radiates 0.1 W of power. Regarding the results, the researchers observed healthy and osteopenic/osteoporotic states across 6 participants (23–94 years old, 48 women, 12 men). Nonetheless, that study employs a licensed frequency band (except for the range between 902 and 928 MHz, the lowest ISM band). That is, typical commercial applications, such as analog and digital TVs or even signals from a simple cell phone, represent interfering signals and can cause measurement errors. Hence, one aspect to consider is the necessity of electromagnetic shielding surrounding the antenna pair's emitting and receiving space, which was not referred to in the article.

Additionally, regarding [11], it should be underscored that, since the radiofrequency device is contained in a protective enclosure, the S-parameters cannot be calibrated and measured directly at the power splitter ports. Therefore, the microwave device was calibrated along with the cables running to the network analyzer. This leads to the oscillatory behavior of S-parameters, and to eliminate these spurious oscillations, de-embedding was performed after measurements. Furthermore, despite the designated region being mainly composed of bone tissue, in patients with a high percentage of fat, the other tissues may be relevant, to the point of altering the measurements. Last, to definitively validate the device's measurements, it is essential to compare them with an existing test (preferably DXA because it is the gold standard) under the same conditions (gender, age, body fat, etc.). This will probably result in the need to process the measured signals to introduce other variables for the expression proposed in the study. The intent is to obtain a final result for each diagnosis that is reliable and more accurate.

Another study developed using EM wave techniques for osteoporosis diagnosis was proposed [10] to qualify the relative electrical permittivity of bone tissue. In this vein, it sought to identify values compatible with healthy subjects and those with low BMD. The method is based on measuring the middle phalanx of the middle finger with an antenna positioned on one side and a reflective surface on the other. The reported results use a previous test of patients' bone densitometry (DXA) as a reference. In addition, considering that a very porous bone tissue characterizes a lower permittivity than a healthy tissue, the researchers proposed scores for frequency deviation values (concerning the resonance frequency of the antenna used in the diagnosis) to classify the patients as healthy, having low BMD, or having osteoporosis.

However, as mentioned in [10], some significant parameters, such as finger dimensions, are not yet considered. Moreover, no differences regarding patients' age, gender, fat percentage, or any other potentially relevant aspects were mentioned in the study. Additionally, no reference was made to electromagnetic protection to confine the signals used in the diagnosis, which would arguably prevent external interferences from modifying the values obtained.

In [12], after bone mineral density measurements in the middle phalanx of the middle finger using AccuDEXA (a device that uses DXA), the researchers found a correlation with DXA measurements in the spine, hip, or forearm. This reinforces the possibility of using this site to identify patients with low BMD.

*Osseus* is currently undergoing validation for use in human subjects, with ethics committee approval (CAEE 39675020.0.0000.5292, HUOL/UFRN), through tests that assure the device's accuracy regarding the gold standard, DXA, in supporting the diagnosis of osteoporosis. The results might be influenced by a survey conducted with patients, including risk factor variables and anthropometric measurements of the middle phalanx of the middle finger, where scanning is performed. A tablet will be used to perform a survey on risk factors, and the data will be preprocessed and stored in the cloud for further analysis. The device has an LCD that, in the future, may also be used to conduct the survey.

The intelligent analysis local system will run the models using as inputs only the individual's data (models to be periodically trained in the cloud and updated in *Osseus*) and the global intelligent data analysis system (cloud) will be more focused on training prognostic models using data from many individuals to identify risk factors, their progression and therefore early detection. Artificial intelligence application proves to be suitable for the prognosis of the disease or fracture since there is no need to provide a diagnostic criterion to identify osteoporosis but a set of examples that represent the variations of the disease. Additionally, a number of classification algorithms and methods have been applied to machine learning problems [13–15]. However, it has been observed that it takes more than merely choosing an algorithm and running it over the data for such learning to be successful. That is, many learning patterns can have a diversity of parameters. Such patterns must therefore be appropriately selected for better classification results to be obtained.

Thus, there is a lack of a unified system with different databases and grouping the various attributes and clinical factors to provide broader coverage of different populations when identifying risk groups. For instance, these factors should include male participants, broader age groups, and race. Risk factors are of valuable help in identifying groups with a potential for osteoporosis. However, when individually analyzed, such factors are insufficient to track the groups. Therefore, it is necessary to cross-reference information from previously performed tests and then use an artificial intelligence tool for treating data.

AI and, in particular, neuronal networks have demonstrated potential for diagnostic/classification problems in the clinical area and in particular in osteoporosis [16–19]. In addition, it is expected to be able to identify the risk factors that most influence the progression of the disease and, thus, be able to develop models for early detection.

## Conclusions

Predicting the risk groups for osteoporosis may mitigate the substantial financial burden of osteoporotic fractures on health care systems. Considering that the pattern of change in BMD with age is reasonably well understood and that the nonexistence of an independent contribution of BMD to fracture risk is necessary to associate it with somatic factors, several methods make use of the integration of artificial intelligence with risk factors to screen groups at higher risk for osteoporosis or fractures. Given that screening is intended to direct interventions for those necessitating the diagnosis of osteoporosis, the tests must be of high specificity. In this context, the most significant variables were chosen for a survey to be performed with humans for testing in future work.

Finally, another field of activity in our project is the development of progressively smaller *Osseus* devices with greater portability and lower manufacturing costs. Of note, it will be necessary to carry out tests using different artificial intelligence approaches, owing to the complexity of the data that this tool will have to deal with throughout clinical trials. Therefore, it will be possible to have a predictive tool that can be applied to populations with more individuals—including males—since most studies focus on females, regardless of their age or ethnicity.

## Methods

### Antennas

An antenna is a device that beams EM waves into free space. Typically, the antenna is powered by a transmission line, such as a microstrip line or a coaxial cable, which transmits signals from the transmission source to the antenna [20]. Over the past few years, several types of medical applications that use antennas have been thoroughly investigated and reported, including the diagnosis and treatment of different chronic diseases [21]. Among the existing types, the Yagi-Uda antenna, or simply the Yagi antenna, features high directivity and, consequently, high gain in the direction of maximum energy transmission. These factors lead us to choose this type of antenna, This antenna consists of multiple director elements, which conduct the electromagnetic signal to the half-wavelength dipole, the element through which the signal is injected, called the radiator element, and a reflector element placed behind the half-wave dipole. Hence, the half-wave dipole is powered by the transmitter signal, which generates a current distribution along its length, resulting in the transmission of microwaves. Next, given their mutual coupling with the closest element, the director elements guide the microwave due to the current induction generated by the previous elements [20].

**Fig. 4:**
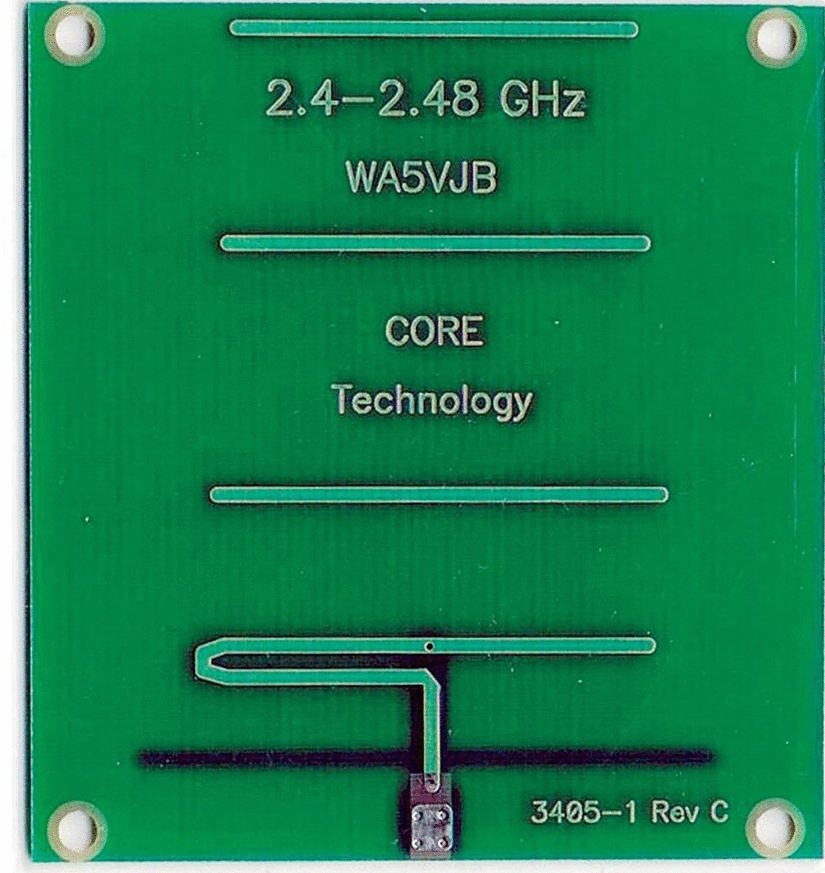
Antenna used on *Osseus*

**Fig. 5:**
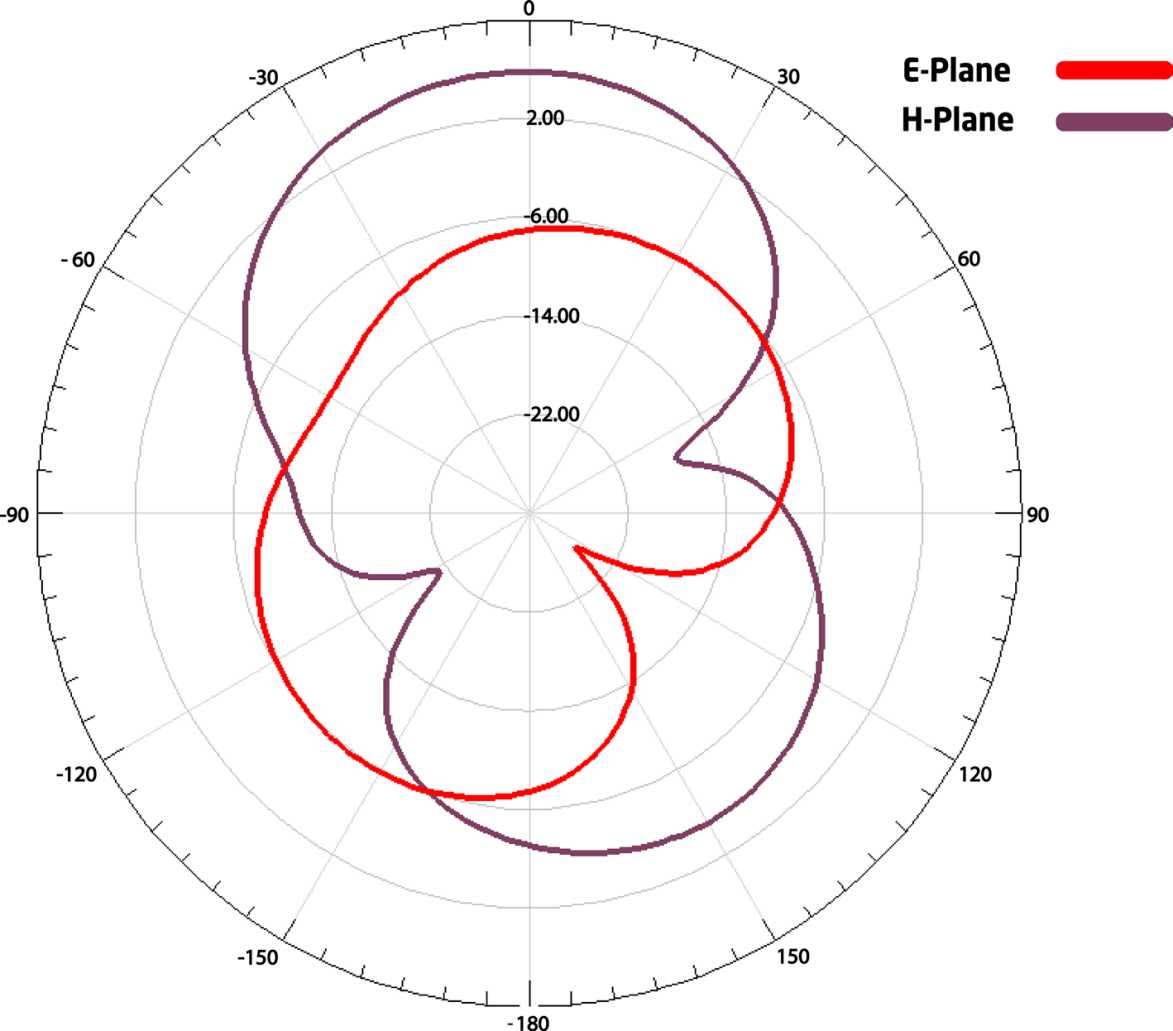
Radiation pattern of the *Osseus* antennas

In this way, the pair of antennas used on *Osseus* corresponds to the model WA5VJB (Fig. 4), marketed by Kent Electronics. The operating frequency is the most common microwave frequency used, that is centred at approximately 2.45 GHz, which lies within the Industrial, Scientific, and Medical (ISM) radio band and is reserved for such purposes [22]. This frequency does not require a license for operation, nor payment of fees due to the use of the electromagnetic spectrum. In addition, the necessary circuits for implementing the electronics of the system, as well as the absorbing foams used in the electromagnetic shielding are easily accessible in this frequency range and at an attractive cost, for large-scale manufacturing. Hence, this pair of antennas was designed to operate in a 2.40–2.48 GHz frequency range, and it has a radiation pattern, as indicated in Fig. 5.

Additionally, it is typical for electronic circuits near the antennas to be sensitive to electromagnetic interference (EMI), and it is necessary to protect them from this effect. The technique used for such a process is called shielding, and it can be accomplished by several means. One of the simplest shielding methods for external interference is to surround the circuit with a metal box, usually made out of aluminum, responsible for reflecting these EM waves and thus preventing them from reaching the circuits.

### Electromagnetic shielding

Shielding can attenuate an electromagnetic wave. Such attenuation occurs through the reflection of the incident wave due to the use of conductive material in the shielding, such as iron, steel, copper, or aluminum, the latter being more versatile and low cost. Then, its efficiency is calculated as a ratio of the power inside and outside the shielding [23].

With the need to protect measurement equipment, applying EM shielding has become relevant, especially in electronic devices used in the medical field. They are composed of sensitive analog amplifiers and microprocessors that can be affected by EM interference, among others.

Internally, there is a need to prevent the signal radiated by the transmitting antenna from reflecting off on the metallic walls of the box and returning to the electronic circuitry. Therefore, it makes it necessary to coat the metal box internally with an absorbing material. Electromagnetic absorbers are composed of materials that absorb the incident microwave in specific frequency ranges and release it as heat. These materials are obtained from the appropriate processing of polymeric matrices that act as absorbing centers for the incident microwave. This study used *Eccosorb AN* microwave absorbers designed to strongly attenuate a specific frequency range, confining the signal within the box and avoiding successive reflections on the metallic walls. Such absorbers are made of polyurethane foam treated with carbon and mounted on a laminated surface to generate a controlled conductivity gradient [24].

### Electronic devices

In regard to *Osseus*, the processor is another significant device. Processors are devices of a few square centimeters in size and with high processing power. They can operate analog-to-digital conversions, with the ability to deliver results in a graphical environment over a computer network, perform data analysis, and compute them in a neural network by receiving programming instructions.

To generate the targeted frequency, we used a voltage-controlled oscillator (VCO). This component can generate a sinusoidal signal at a frequency that depends on the voltage applied to its input, and it is located at the transmitter side of the signal. On the opposite side, an RF power detector is connected to the receiving antenna. The sensor converts the received RF intensity into voltage so that it can be processed.

To obtain the desired directivity, we used a pair of Yagi Uda antennas printed on a fiberglass substrate, with dimensions of 6.6 cm × 7.0 cm, one being a transmitter and the other a receiver, operating at the frequency range of 2.45 GHz [25], fed by a microstrip line. After the prototype's three-dimensional modeling, we proceeded with the fabrication of the components. Part of the device was manufactured with 1.6 mm thick aluminum, ensuring a high standard of EM shielding. The front part was built with translucent acrylic and the remaining components were 3D printed. On the inside, the equipment was coated with absorbing material designed for a 2.45 GHz frequency.

### Proof of concept

We performed simulations to prove the operation of the device using samples of orthodontic plaster cylinders simulating human bone encased in porcine skin. This kind of skin has been recommended for studies since it has physiological, histological, and biochemical similarities and density close to that of human skin [26]. Each plaster cylinder was built with only water and plaster, 2 cm in diameter and 7.5 cm in height.

**Fig. 6:**
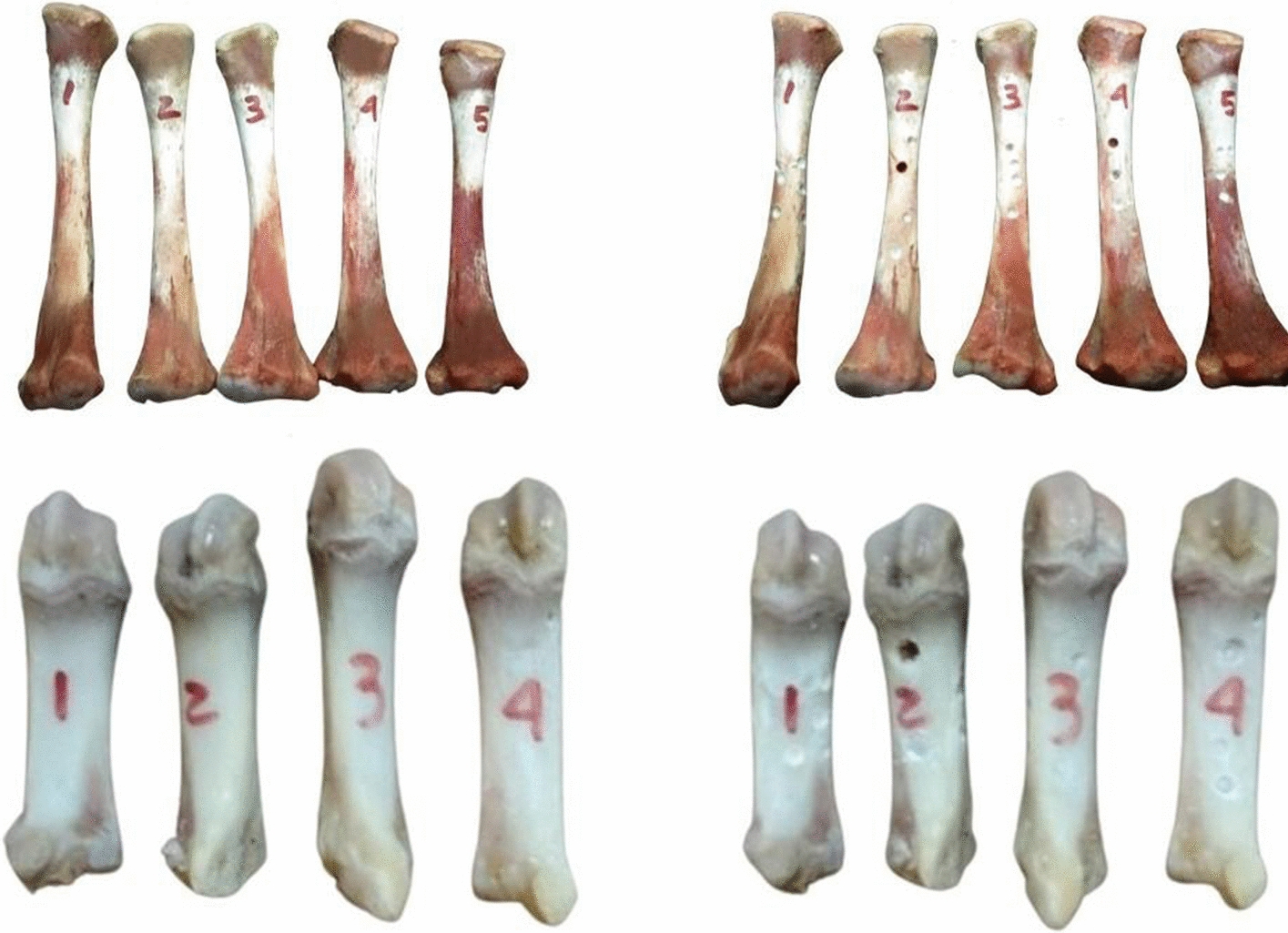
Intact and altered samples of poultry and porcine bones used for proof of concept

Next, we used the femur poultry bones since, in addition to representing the first bird order associated with humans, such birds have relatively long femur bones [27]. Moreover, we also used bones from the porcine proximal phalanx, given their remarkable similarity to human bone tissue [28] and bones from cattle femurs since they satisfactorily reproduce aspects of human anatomy [29]. First, the samples were classified and analyzed while still intact (original sample) and after undergoing mechanical perforations to artificially increase porosity (altered samples—Fig. 6). According to the perforations, the samples have undergone modifications in their attenuation measurements.

In order to verify the correlation between the intensity of signals measured—after the beams passed through the samples—and bone density, several samples were selected and tested in this study. First, the device’s circuit was activated in a way there was no obstacle between the antennas for maximum signal strength in the receiving antenna, and the value it measured was recorded. Then, we placed each sample individually between antennas, the circuit generated and propagated the signal from the transmitting antenna, and then it was captured and recorded.

The signal received by the antenna is converted into a digital signal using an analog-to-digital converter, and at each reading request, 50 readings are actually taken, which are stored and sorted for removing outliers.

The following formula was applied for calculating the quartile:

$${Q}_{j}={X}_{k}+ \frac{(j\mathrm{th}(n+1)}{4}-k ({X}_{k+1}-{X}_{k})$$

where *Q*_*j*_ is the *j*th (from 1 to 3), *X* is the set with *n* readings and *k* is the position of the median. Then, the interquartile range (IQR) is calculated by using:

$$\mathrm{IQR} = {Q}_{3}-{Q}_{1}$$

Thus, the lower limit of the first quartile and the upper limit of the third quartile can be calculated with the two equations:

$$\mathrm{lower} = {Q}_{1}- 1.5 \times \mathrm{IQR}$$

$$\mathrm{upper} = {Q}_{3}+ 1.5 \times \mathrm{IQR}$$

Finally, all elements of the set that are within these limits are selected and the arithmetic mean is then calculated, which will be displayed as the result of the received signal strength reading.

The "difference" column is the result of the following formula:

$$\mathrm{Dif}={\mathrm{At}}_{\mathrm{o}}-{\mathrm{At}}_{\mathrm{a}}$$

where Dif is the difference between the values, At_o_ is the attenuation value of the original sample (without physical alterations), and At_a_ is the attenuation read after the sample was mechanically changed.

## Data Availability

All data generated or analysed during this study are included in this published article and its supplementary information files.
